# Is Copper Still Safe for Us? What Do We Know and What Are the Latest Literature Statements?

**DOI:** 10.3390/cimb46080498

**Published:** 2024-08-02

**Authors:** Angelika Edyta Charkiewicz

**Affiliations:** Department of Clinical Molecular Biology, Medical University of Bialystok, 15-269 Bialystok, Poland; angelika.charkiewicz@umb.edu.pl

**Keywords:** copper, toxicity, use of copper, natural concentration of Cu, healthy/harmful aspects of copper

## Abstract

Copper (Cu) is a precious metal and one of the three most abundant trace elements in the body (50–120 mg). It is involved in a large number of cellular mechanisms and pathways and is an essential cofactor in the function of cellular enzymes. Both its excess and deficiency may be harmful for many diseases. Even small changes in Cu concentration may be associated with significant toxicity. Consequently, it can be damaging to any organ or tissue in our body, beginning with harmful effects already at the molecular level and then affecting the degradation of individual tissues/organs and the slow development of many diseases, such as those of the immunological system, skeletal system, circulatory system, nervous system, digestive system, respiratory system, reproductive system, and skin. The main purpose of this article is to review the literature with regard to both the healthiness and toxicity of copper to the human body. A secondary objective is to show its widespread use and sources, including in food and common materials in contact with humans. Its biological half-life from diet is estimated to range from 13 to 33 days. The retention or bioavailability of copper from the diet is influenced by several factors, such as age, amount and form of copper in the diet, lifestyle, and genetic background. The upper limit of normal in serum in healthy adults is approximately 1.5 mg Cu/L, while the safe upper limit of average intake is set at 10–12 mg/day, the reference limit at 0.9 mg/day, and the minimum limit at 0.6–0.7 mg/day. Cu is essential, and in the optimal dose, it provides antioxidant defense, while its deficiency reduces the body’s ability to cope with oxidative stress. The development of civilization and the constant, widespread use of Cu in all electrical devices will not be stopped, but the health of people directly related to its extraction, production, or distribution can be controlled, and the inhabitants of nearby towns can be protected. It is extremely difficult to assess the effects of copper on the human body because of its ubiquity and the increasing reports in the literature about its effects, including copper nanoparticles.

## 1. Introduction

Copper (Cu)—*Cuprum* is a chemical element with an atomic number of 29. It is currently estimated that the level of demand in production for this element will virtually double by 2030, in line with current demand. It is one of the three most useful and necessary metals in the world, after iron and aluminum [1,2,3,4,5]. This is likely to be influenced by the tremendous progress in both research work and the milestone development of technological innovations, which is the main focus of many researchers as well as production sources.

For many years, it has been used by practitioners of unconventional medicine (radiesthesists), for example, to make at least jewelry (bracelets, brooches, etc.) or talismans/amulets. Meanwhile, in traditional medicine, it plays a rather important role, including facilitating the incorporation of iron into hemoglobin. Both its excess and deficiency are harmful [6,7,8,9]. This article presents a summary of its action in a multifaceted health approach as well as its comprehensive use. It represents an innovative approach to the analysis of this element. 

**Figure 1:**
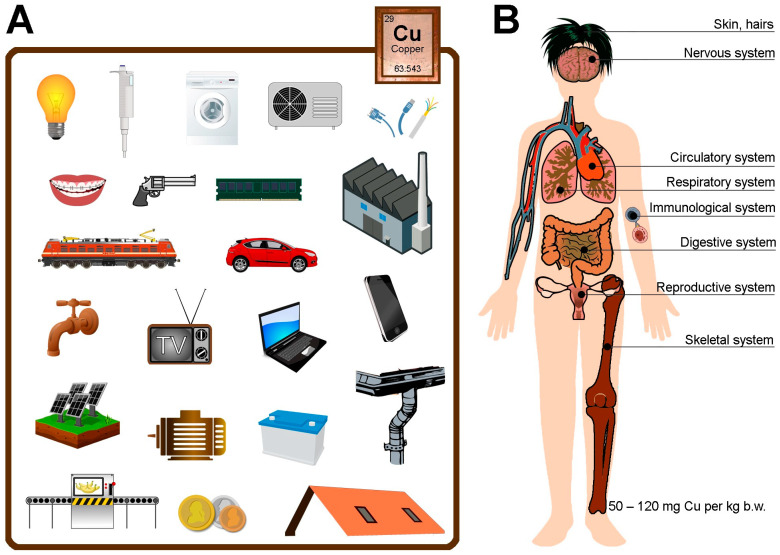
Sources of copper and its most significant effects on different parts of the human body.

As it is unquestionably a very good and powerful antioxidant, it is necessary to emphasize its other side as well, for it can be toxic, and it is omnipresent in practically every device. Consequently, it can be damaging to any organ or tissue in our body, be-ginning with harmful effects already at the molecular level and then affecting the degradation of individual tissues/organs and the slow development of many diseases, such as those of the immunological system, skeletal system, circulatory system, nervous system, digestive system, respiratory system, reproductive system, and skin (Figure 1).

Figure 1 illustrates some of the most common sources of Cu (A) and how exposure to Cu leads to the development of many diseases or pathologies in the body (B). It also shows the health effects associated with the long-term accumulation of Cu in organs/tissues and its reaction in the body.

The main purpose of this article is to review the literature with regard to both the healthiness and toxicity of copper to the human body. A secondary objective is to show its widespread use and sources, including in food and common materials in contact with humans. 

## 2. Functions of Copper in the Body

This trace element is found in very small amounts in tissues or cells, while it is most abundant in bone and liver [6,10,11,12,13]. It is considered a major player involved in a large number of cellular mechanisms and signaling/cellular pathways, taking part in oxidation as well as reduction processes. It is involved in the neutralization of free radicals as well as altering the sensitivity of cells to their toxic effects [14,15,16,17]. It is also an essential cofactor in the function of cellular enzymes such as cytochrome oxidase, catalase, dopamine-beta-hydroxylase, and peroxidase [5,11,18,19,20]. It supports the work of many energy-producing enzymes, oxygen metabolism—mitochondrial respiration (cytochrome oxidase (COX)), also building red blood cells, collagen, or connective tissue—collagen cross-linking (lysine oxidase (LOX)), skin pigmentation (tyrosinase), neurotransmitters in the brain (catecholamine biosynthesis (dopamine b-monooxygenase)), antioxidation (zinc–copper superoxide dismutase (SOD)), and, for example, facilitating the absorption or breakdown of iron [6,10,18,19]. It is an important catalyst for heme synthesis and iron absorption and is the third most abundant trace element in the body (after Zn and Fe) [5,13].

Its first deficiencies in humans were documented in the 1950s, where it was shown that a deficiency of this pierivirine causes hypocopperemia, hypoceruloplasminemia, and neutropenia in infants [7,19]. The extent to which it is absorbed depends on its chemical form and the copresentation of other dietary components, lifestyle, or disease entity. It is most readily absorbed with reduced glutathione (GSH) and organic acids (citric, gluconic, lactic, acetic), while its absorption is impaired by zinc, iron, calcium, molybdenum, phosphorus, and vitamin C. Most (30–50%) is absorbed in the small intestine (Cu^2+^) due to transport with albumin or transcuprein, and least in the stomach and digestive juices [7,8,20,21]. The human body contains 50–120 mg of copper (0.79 to 1.9 mmol) [12,21,22,23]. The liver stores (18–45 μg Cu/g dry weight) Cu in hepatocytes, which it secretes into the plasma or with bile. Copper is removed via the biliary route (80% from the liver), with feces (80–90% from the diet; 2 mg/day), sweat (50–100 μg/day), urine (10–50 μg/day), and very little with hair. Approximately 60–90% of Cu circulating in the blood is in the form of ceruloplasmin (an antioxidant), transporting it to tissues with histidine, albumin, or transcuprein. Blood contains about 6 mg Cu, and its homeostatic regulation protects the body from both toxicity in the case of increased absorption or Cu deficiency in the case of increased excretion. The upper limit of normal in serum in healthy adults is about 1.5 mg Cu/L, and the safe upper limit of average intake is set at 10 mg/day for women and 12 mg/day for men. Cu poisoning most commonly results in weakness, lethargy, anorexia, erosion of the gastrointestinal epithelium, gastrointestinal disturbances, and hepanism [5,7,21,22,23]. As recent reports suggest that both copper excess and deficiency can be harmful, it is important to carefully monitor homeostasis, especially in neurological and cardiovascular diseases and liver status [6,15,20]. This element accumulates (Table 1) most often in the liver, kidneys, lungs, bones, muscles, brain, adipose tissue, and skin, inhibiting the activity of many enzymes (Figure 1) [6,9,11,13,14,16,17,18,19,22,24,25,26,27,28,29,30,31,32,33].

### 2.1. Immunological System

It is an element required for the proper functioning of the immune system and owes its antioxidant properties to its occurrence in numerous metalloenzymes. It also has a protective effect on DNA, proteins, or lipids [6,18,24]. It can catalyze the production of hydroxyl radicals in a Fenton-like reaction, unfortunately causing oxidative stress and cell damage, subsequently affecting protein aggregation processes or inflammation of the nervous system [6,17]. Cu shows an easy transition between oxidation levels +1 and +2 in biological systems, and the very mechanism of action of these complexes is based on their redox activity and the induction of reactive oxygen species (ROS), which can lead to lethal oxidative stress [34]. It is noteworthy that its deficiency significantly affects the development and function of the immune system, including humoral and cellular immunity and the production and secretion of immunoreactive substances [11,13,19,20,35,36]. Cu homeostasis is essential for healthy brain development and function, which is required for certain enzymes responsible for various metabolic processes in various tissues, including the brain. It is a cofactor for antioxidant enzymes (e.g., superoxide dismutase and glutathione peroxidase) that provide protection against ROS damage [36]. Among the most common symptoms of this deficiency are increased host susceptibility to various pathogens, reduced numbers and impaired function of neutrophils, reduced proliferation of splenocytes, reduced antimicrobial activity of macrophages, impaired ability of B lymphocytes to produce antibodies, and impaired function of cytotoxic T lymphocytes and helper T lymphocytes [19]. Still, its actual mechanism in many immune processes is not fully defined and understood [35]. Its antimicrobial toxicity is directly exploited by phagocytic cells to kill pathogens (bacteria and fungi by macrophages) that accumulate at sites of infection [11,13]. Its main function is the constitution of enzymes that transfer electrons (oxidases) to reduce molecular oxygen. It is essential for energy metabolism at the cellular as well as molecular level [21]. Copper, including copper nanoparticles, can help in broad-spectrum antiviral therapies, including with bronchitis virus, polio, flu, herpes virus, human immunodeficiency virus (HIV-1), and even SARS-CoV-2 [37,38]. Copper nanoparticles can interact with the thin bacterial cell wall, DNA, nucleic acid, etc. They are also responsible for the production of ROS and oxidative stress, ultimately leading to cell death [39]. A new Cu-ion-induced cell death pathway recently discovered by Tsvetv et al. [40] has been named “cuproptosis”. It is a form of metal-regulated cell death induced by this ion, which induces apoptosis-independent cell death through direct binding to TCA (lipoylated tricarboxylic acid cycle) proteins. Importantly, by then inducing the aggregation of lipoylated proteins and destabilization of Fe-S cluster proteins, it leads to proteotic stress [40].

There is no doubt that the effects of copper deficiency on the immune system in humans require further and more detailed studies.

### 2.2. Skeletal System

Copper is involved in strengthening connective tissue structures, including bones, as it is mainly found there. The level of copper in the blood is correlated with the maintenance of normal bone density and a reduction in the risk of fractures, which in the long term is particularly important in menopausal women [10,22,25]. A value of 5–6 mg/kg sm has been recognized as the physiological level of Cu in bone tissue (its levels vary from 0.16 to 6.30 mg/kg dry weight). Its concentration is highest in the ribs and lowest in the tibia and decreases with age [22]. There are a number of publications and studies confirming the efficacy of copper supplementation (approximately 3 mg per day), resulting in a reduction in the decrease of bone mineral density, characteristic especially during menopause. In elderly patients, regular supplementation may lead to an increase in bone resorption markers such as NTX C (collagen type I cross-linked C-telopeptide) or DPD (deoxypyridinoline) [22,41,42]. Meanwhile, in children, it may result in the reversal of adverse bone changes [43] and have a beneficial effect on joint health [26]. Nevertheless, there is a lack of studies confirming osteoblastic and osteoclastic activity, as well as bone resistance to copper [21,22]. In an analysis by Randanelli et al. [21] on the effect of copper supplementation (2.5–3 mg/day) and its effect on bone metabolism, it was shown that there is a slowing of mineral loss from bone and thus a reduction in resorption rates. Copper stimulates the differentiation of mesenchymal stem cells toward the osteogenic lineage [22,44]. Meanwhile, its severe deficiency due to either low dietary intake or a possible mutation present in the body is associated with weakened connective tissue [20]. Both the effective homeostatic regulation of absorption and retention help protect the body from toxicity as well as copper deficiency and are mainly inhibited by the presence of phytylones, calcium, ascorbic acid, and other trace elements. Interestingly, copper has been shown to have positive effects on cells that regulate bone metabolism and copper ions that can inhibit osteoclastic resorption [21,45]. It is worth noting that low concentrations of Cu improve the viability and growth of osteoblastic cells, while higher concentrations can be cytotoxic [21,46]. In addition, Cu has been shown to stimulate differentiation of mesenchymal stem cells toward the osteogenic lineage [44]. Also, copper deficiency impairs the strength of the mechanical tissues of the bones, due to reduced elastin and collagen networks [45].

### 2.3. Circulatory System

Copper has a significant effect on the functioning of the cardiovascular system due to its high antioxidant potential. It supports the function of the heart and blood vessels through the synthesis of red blood cells, improving blood morphotic parameters. It also minimizes the risk of developing atherosclerotic lesions by reducing plasminogen activator inhibitor type 1 (PAI-1) levels by up to 30% with regular supplementation (6 mg/day) [22,27]. Its increased levels can accumulate in the myocardium, adversely affecting its function, both in terms of possible deficiency and toxicity [5,10,30]. In hieprlipidaemic patients also supplementing with copper, a reduction in total and LDL cholesterol and triglycerides has been observed, with a concomitant increase in HDL cholesterol [5,10,47]. Copper also indirectly affects the efficient synthesis of hemoglobin and erythrocytes, aiding in the prevention of iron deficiency anemia, the most common cause of anemia [48]. Its release in large quantities by the liver causes its rapid accumulation in erythrocytes and subsequent oxidant production affecting red blood cell damage [5]. To date, no direct effect on cardiovascular mortality has been confirmed in individuals with higher or lower blood levels, so further research in this direction is important [10]. In the meantime, serum copper levels have been linked to the risk of atherosclerosis in middle-aged men, although a significant potential link to venous thromboembolism (VTE) is still missing [30]. Meanwhile, severe copper sulphate intoxication can cause intravascular hemolysis (anemia, increased plasma hemoglobin concentration, reticulocytosis, hemoglobinuria, hyperbilirubinemia, wrinkled and fragmented erythrocytes, formation of Heinz bodies, among others) [5,12]. There is no evidence to support an association between copper concentrations and CVD risk or the effect of copper supplementation on CVD [12]. Copper deficiency in myocardial cells can lead to impaired myocardial energy consumption, reduced ability of the heart to contract and induce cardiomyopathy, and the accumulation of catalytically active Cu^2+^ in cardiac extracellular muscle induces the toxicity of this element, which is suggested to be an important catalyst for cardiovascular damage. It can also lead to increased mitochondrial density, disruption of the crest, and other unapplied myofibrils with disruption of Z-bands in cardiomyocytes. Interestingly, both copper deficiency and excessive copper accumulation can induce cardiac hypertrophy, leading to redox imbalance through its effects on mitochondrial respiration, protein metabolism, and lipid metabolism. Ultimately, it can induce hypertrophy or loss of these cells [49].

Individual copper nanoparticles are able to move between cells or penetrate cell membranes and eventually enter the circulatory system, spreading from the initial site of exposure to different parts of the body and eventually accumulating in organs [39,50].

Despite the available laboratory tests, more clinical studies need to be conducted, taking into account a range of information about the patient themself.

### 2.4. Nervous System

Copper supports normal brain development and immune function. It is a component of superoxide dismutase and an antioxidant enzyme that helps eliminate harmful oxygen “free radicals” [10,16]. Copper, through its protective effects, has the effect of reducing the levels of beta amyloid in the cerebrospinal fluid, an abnormal protein whose cerebral deposits are found in Alzheimer’s disease. This contradicts previous studies suggesting just the opposite effect of this element on the course of Alzheimer’s development. Meanwhile, long-term supplementation (8 mg/day) may have a positive effect on the progression of the disease, as well as, interestingly, improving motorial function but in the course of Parkinson’s disease [7,28,51]. Unfortunately, there are still controversial observations regarding dietary copper deficiency versus the etiology and pathophysiology of Alzheimer’s disease [12,52]. Some studies show that people with higher copper levels have a lower risk of Alzheimer’s disease [53], while others show that excess dietary copper is involved in the development of the disease [54]. Unfortunately, its accumulation already in damaged brain regions in this disease may not directly show overall copper status or copper intake [55]. Copper plays a role in the onset of Alzheimer’s disease, as the hippocampus, cerebral cortex, cerebellum, and brainstem are also pathways for the overall progression of the disease [56,57,58]. As it is important in controlling gene expression in the nucleus accumbens, it is also involved in brain myelination. And by modulating synaptic activity, as well as excitotoxic cell death and signaling cascades induced by neurotrophic factors, it is important for a wide variety of neuronal functions [6,10]. It should be noted that copper stimulates brain function and modifies neural transmission and has a positive effect on cognitive function. Meanwhile, in school-aged children, its higher levels in the body are associated with, among other things, better memory, concentration, or cognitive techniques [6,20,59]. Copper is also actively involved in mitochondrial synthesis including bioenergetics, dynamics, and mitophagy and how this influences cell fate through metabolic reprogramming [29]. The very mechanism in which Cu is involved in the development of neurodegenerative diseases is not yet fully defined, and oxidative damage in the brain may result from interactions between Cu and homocysteine, or amino acids containing a thiol group. Increased Cu concentrations and/or homocysteine levels in the elderly may promote significant oxidative damage to neurons and may cause Alzheimer’s disease or another neurodegenerative disease (Parkinson’s or amyotrophic lateral sclerosis) [6,7]. Copper plays an important role in the physiological procs of the brain, although it has not yet been fully elucidated [6]. An inadequate intake of this element, or an existing mutation in the body, may be associated with mental retardation or even impairment of central nervous system (CNS) function [20]. It is worth to mention, first of all, that copper nanoparticles can contribute to the progression of neurodegenerative diseases, including Alzheimer’s and Parkinson’s diseases [37,60].

### 2.5. Digestive System

The occurrence of chronic Cu toxicity may mainly affect the liver, manifesting as liver cirrhosis with episodes of hemolysis and damage to the renal tubules, brain, and other organs. As a further consequence, this can lead to coma, hepatic necrosis, vascular collapse, or even death [8,20]. Copper from the diet can be partially absorbed in the stomach. Its strongly acidic environment then releases bound copper ions from partially digested food particles. The largest part of ingested copper passes into the duodenum and ileum, the main site of absorption. Much of it is soluble in the digestive tract due to complexation with amino acids, organic acids, or other chelators. Meanwhile, its ions enter the cells of the mucosa lining the intestine by simple diffusion, and then escape to the basolateral surface by another means of transport via copper-transporting adenosine triphosphatase (ATPase). Copper circulating through the body returns to the liver bound to cerulopasmin and is then secreted into the blood with it [20].

A range of up to approx. 1.5 mg/L serum copper concentrations are present in healthy people, and up to approx. 3 mg Cu/L in people with gastrointestinal symptoms. Meanwhile, ingestion of liquids containing concentrations >30 ppm Cu causing multiple gastrointestinal irritations (nausea, vomiting, abdominal pain), or drinks/foods containing ≥25 mg Cu/L are associated with acute gastroenteritis. Renal dysfunction is usually mild and may develop more like anuria or scanty urine [5]. In contrast, consumption of contaminated food or water may result in the development of acute gastrointestinal symptoms [5,7].

An excess of copper occurs very rarely and occurs mainly in the case of its continuous storage and as a result of feeding boiling liquids from corrosive copper or brass vessels, similar to lead or cadmium [10,61,62]. Either accidental or intentional copper overdose can cause acute liver damage. And chronic consumption of excessive amounts may result in copper overload in the body and chronic liver damage [12]. In the case of recommended oral or parenteral supplementation (e.g., in Wilson’s disease), its close monitoring in the body is important [13]. In Wilson’s disease, where there is a pathogenic accumulation of hepatic copper that is a source of ROS [12,63,64], copper absorption from the diet is not usually controlled by food absorption but is regulated by copper excretion in the bile. Copper is present in many foods, and its limitation has long been considered an important aspect of treating Wilson’s disease. Copper, when it is properly absorbed in the intestine by enterocytes, is transported with proteins via albumin or transcuprein to the sinusoidal plasma membrane in the liver. Copper atoms are then transported into hepatocytes by the copper transporter copper transporter 1 (CTR1) into the cytosol, and in turn, copper chaperones (e.g., superoxide dismutase (CCS), antioxidant protein 1) transport copper to specific intracellular targets. It is noteworthy that if intracellular copper levels are high, then ATP7B moves its cellular compartment to bile-duct-associated structures facilitating the process of the excretion of copper into the bile ducts [64,65].

It is worth emphasizing that copper deficiency in the serum or liver also occurs in many other liver diseases and is very rarely reported by these patients [13]. There is also no doubt that understanding the nature of copper deficiency in liver diseases requires further research and a more holistic understanding of the occurrence of this element in the environment.

### 2.6. Respiratory System

There are still few data on the absorption of copper into the lungs. One of the most common symptoms is the occurrence of fever caused by metal fumes (chills, muscle aches, headache, malaise, dry throat) when welding copper alone. Then, during the volatilization of metallic copper, the lungs only absorb a certain amount of copper, and it is low due to the high temperatures required for volatilization. It then irritates the respiratory tract, mainly the mucous membranes of the mouth, eyes, or nose. Also, its intensive and regular inhalation may cause perforation of the nasal septum [5,8]. Copper ions quickly enter the circulatory system after inhalation [23]. It should be noted that dysregulation of Cu homeostasis can lead to immune dysregulation, including oxidative stress, followed by chronic inflammation and angiogenic disorders, which may consequently contribute to respiratory diseases. As pointed out by Song et al., the pathological effects of Cu in respiratory diseases mainly include immunity, oxidative stress, chronic inflammation, and angiogenesis [65]. Larger amounts are inhaled in the vicinity of large smelters [8]. In a cohort study of ~0.8 million adults in Toronto, Canada it was shown that long-term exposure to copper in PM_2.5_ (fine particulate matter) and estimated ROS (reactive oxygen species) concentrations in lung fluid were associated with an increased incidence of respiratory disease. This suggests adverse effects of emissions from outside the tailpipe (e.g., brake/rail wear and engine wear) on the respiratory system. An association between Cu and COPD mortality was shown in this long-term study, although this association or increased morbidity was not demonstrated for asthma [66], POChP, PAH and respiratory tract infections, lung cancer, and interstitial lung disease [65]. Meanwhile, Denmark showed no association between the element analyzed and cardiovascular mortality [33], while the Netherlands only showed a worsening of lung function [67]. According to Janssen et al. [23], there is also a potential link between tobacco inhalation and the potential depletion of copper ions in the lungs. The authors speculate that inhalation copper therapy may be used as one treatment option for patients with emphysema. Meanwhile, inhaled copper monotherapy may be associated with fibrosis formation through collagen accumulation [23]. Unfortunately, there are still a small number of studies conducted in humans, which does not mean that it should be ignored. In fact, there is a strong rationale for continuing a long-term study, due to increasing environmental pollution [66].

It is worth adding that the new discovery of cuproptosis will certainly contribute to a deeper understanding of Cu metabolic diseases and their molecular mechanisms, no doubt. Merely understanding the mechanisms of Cu metabolism and this discovery will help usher in the future screening of drugs to treat metabolic diseases in humans. Undoubtedly, further experimental and clinical studies are needed to guide therapy and understand the effects of Cu on respiratory diseases [65].

### 2.7. Reproductive System

There is still insufficient work and research on how circulating Cu may be directly related to fertility in humans [16,68,69]. Interestingly, its serum concentration and placental tissue content in women with spontaneous abortion were also assessed. In that case, a significantly higher tissue content was observed in women with spontaneous miscarriage in relation to pregnant women, while a significantly lower serum concentration was observed in women with spontaneous miscarriage in relation to healthy pregnancies [70]. Toxic elements (e.g., cadmium, lead, mercury) can potentially affect reduced fertility [61,62,71,72,73]. Meanwhile, these trace elements (zinc, copper, selenium) are important for maintaining reproductive health in both sexes [16,31,70]. Copper causes infertility in both sexes through a variety of mechanisms, such as changes in sperm motility, reduced sperm quality, or the effect on the egg [73,74]. In a multicenter, prospective cohort study (Screening for Pregnancy Endpoints—SCOPE), unborn women with singleton pregnancies from Adelaide (Australia), Auckland (New Zealand), Cork (Ireland), Leeds, London, and Manchester (UK) were included (*n* = 5628). It was then shown that there was no association between serum copper concentration (30.3–30.5 µmol/L) and time to pregnancy [16]. Positive as well as negative associations were also shown between this micronutrient in follicular fluid in both women with and without endometriosis, a common cause of infertility [75]. It is worth noting that it plays a very important role in male fertility, as it is essential for the production of male gametes. It plays a very significant role in cell division processes—mitotic and meiotic. Large amounts of it are found in the sperm-associated fluids in the epididymis and prostate. Both its deficiency and excess causes reduced fertility in men. It is associated with a whole spectrum of abnormalities at the level of spermatozoa, male gonads, hormone production, and the distribution of, for example, zinc and iron [17,18,31,68,69,76]. High concentrations of Cu in seminal plasma are associated with sperm DNA damage, and since it is essential for healthy spermatogenesis, it can cause toxicity and detrimental effects on sperm quality even at higher doses [68]. It is worth noting that some proteins involved in copper homeostasis (e.g., import pump CTR1) are contained in germ cells and mature spermatozoa, which suggests the important role of this element in both the development and function of spermatozoa [77]. Further studies are very important to confirm the impact or potential impediments to pregnancy or even the process of spermatogenesis itself in copper deficiency/excess. Importantly, attention should be paid to its concentration and the possible occurrence of malformations in children.

### 2.8. Skin

Copper is a component of tyrosinase, a metalloenzyme involved in the synthesis of melanin (skin and hair pigment) [9]. Through its appropriate content, it can prevent premature graying of hair and alleviate the symptoms of vitiligo or show a protective effect against harmful UV radiation. In addition to its beneficial antibacterial action, its beneficial feature is its participation in the synthesis of collagen and elastin. Consequently, it reduces fine lines and wrinkles in the face and, more importantly, accelerates wound healing [9,32,78]. It also improves the overall self-perception of the skin [9,32]. Also, few data are available on the absorption of copper compounds through the skin, as only long-term exposure to copper sulphate or copper azide can be absorbed by the skin [9]. Severe copper deficiency can result in skin loss or change in hair color [21]. Most often, increased copper adsorption is observed in the hair shaft causing a green tinge, especially in damaged parts of the hair. Meanwhile, additional changes also include epidermal loss and, in the case of mechanical damage, can form copper rings. Average copper levels can be around 17.7 ppm in the first 3 cm of the proximal end of scalp hair and 11.9 ppm Cu in pubic hair [5]. Our ancestors already used copper varieties (copper sulphate, malachite etc.) as early as approx. 3000 BC for, among other things, the treatment of difficult-to-heal wounds, surgical wounds, vaginal disorders, or other skin diseases. Currently, copper is also used in face creams. The risk of adverse effects as a result of skin contact (both damaged and undamaged) with copper is extremely low, and ointments containing up to 20% metallic copper also have no toxic effects [9]. Nanoparticles also accelerate hair follicle and sebaceous gland regeneration and accelerated wound closure in rats but may offer a new therapeutic approach to accelerate diabetic wound healing [37,79].

### 2.9. Cancers

Emphasizing once again its antioxidant properties, it is important to mention the use of copper in cancer therapy by inhibiting, among other things, the activity of the p53 protein [32,80]. However, this element may also inhibit the growth and proliferation of cancer cells themselves, their metastasis, or the process of angiogenesis [10,16,19,34,81]. Copper plays an important role for several reasons, including promoting the growth of blood vessels supplying the tumor and activating enzymes and signaling proteins used by cancer cells [10,14]. Meanwhile, its levels are higher in aggressive cells, and the targeted blocking of its bioavailability can reduce the energy required for their movement in the body [14]. Currently, some of the new therapeutic strategies available (such as chelators or ionophores) have shown promising results in preclinical studies, and others are already being used to treat [14,34]. Recent reports confirm that abnormal Cu homeostasis can be observed in many cancers and, consequently, its elevated serum and tissue levels, which are correlated with cancer progression [15,34]. Therefore, it is becoming a new treatment strategy to analyze Cu levels in the body and to pay attention to its homeostasis or its possible influence on cancer progression. There are a number of questions regarding the full understanding of its role in cancer. Yet, current analyses and publications show broad prospects for the use of Cu-coordinated compounds as potential therapeutic agents [15,19,34]. It has been shown that the addition of nontoxic concentrations of Cu to healthy and cancer cells can markedly alter changes in cellular metabolism [32,34].

Several reports confirm the correlation between high Cu levels and the metabolic demand of prostate, breast, colon, liver, colorectal, and brain cancers. And importantly, these types of cancers may be sensitive to Cu-modulating therapies [19,34,82,83]. Meanwhile, studies of copper refinery workers have not shown a positive correlation between copper exposure and an increased incidence of lung cancer, possibly due to arsenic contamination of the ores [5]. The first studies analyzing the effects of copper on novae were initiated by de Jorge et al. in 1965 [84]. However, copper ions have been linked to the development of many cancers, including lung cancer, acute lymphatic leukemia, multiple myeloma, and others, in which copper ion levels are significantly elevated. Research over the past 20 years shows that copper ions are involved in the development, growth, and metastasis of cancer through various pathways. Experimental data have shown significantly elevated copper levels in, for example, stage I multiple myeloma, lung cancer, and acute lymphoblastic leukemia [19].

It is noteworthy, however, that Cu has been shown to play a role in inducing an antitumor immune response by regulating the expression of PD-L1 (programmed cell death ligand 1) [19,85]. Understanding the molecular susceptibility of tumors may have important implications for the efficacy of Cu-dependent drug treatments. And, what seems rather important, the depletion as well as overload of this element in the body may be viable therapeutic strategies to be used in cancer diagnosis [19,34]. There is also no doubt that its specific mechanism in tumorigenesis requires further research.

As shown by Borković-Mitićet al., copper in the brain is an essential factor for CuZnSOD. It mediates the oxidative stress response and neurotransmitter biosynthesis [36].

The use of copper nanoparticles in the treatment of cancer, including breast cancer, can cause a significant reduction in the viability of these cells, morphological deformation of cancer cells, increased production of reactive oxygen species (ROS), and loss of mitochondrial membrane potential. As indicated by studies, the uptake of copper (II) oxide nanoparticles induces apoptosis, but these coppers can also inhibit the viability of cancer cells by necrosis [37,38,86]. In Ehrlich ascites cancer, in vivo resulted in a reduction in tumor volume [87], and in the treatment of human pancreatic cancer, in vitro and in vivo (mouse model) [88]. The authors of one study showed that the degradation of DNA molecules is dose-dependent for copper nanoparticles, generating singlet oxygen. They also observed that they had cytotoxic effects on human histiocytic lymphoma and cervical cancer cells, triggering apoptosis [39,89,90]

### 2.10. Copper Transport at the Molecular Level

Each transporter plays an important role, both the very high affinity CTR1, low affinity CTR2, copper chaperones (CCS, Atox1, Cox17, SCO1, and SCO2), and the copper efflux transporters ATP7A and ATP7B. They play a key role in that they are responsible for the uptake, distribution, and export of copper from cells to individual organs or tissue. It is important to understand that cells contain a complex network of soluble regulatory molecules. These enable fine-tuning of the homeostasis of copper itself and the precise temporal and spatial allocation of copper within the cell itself, making it possible to understand the scheme a little better. There is also a strong interaction with dynactin, ADP-ribosylation factor GTPase (Arf)1, phosphatidylinositol-binding protein (COMMD1), the ubiquitin machinery, and other proteins responsible for the metabolism of copper itself. There is a strong need for an in-depth study of the relationship between copper homeostasis and other physiological processes (e.g., lactation, inflammation, lipid metabolism, etc.) to better exploit the therapeutic potential of copper transport regulation in humans in the future [11,38,91]. This molecular mechanism is very complex yet still represents significant interest and probably high therapeutic potential.

## 3. Effects of a Deficiency as Well as an Excess of Copper in the Body

Both the deficiency and excess of Cu can lead to the appearance of numerous disorders in the body (Table 2). The most common causes of deficiency are an improperly balanced diet (e.g., strict vegetarian diet) and various types of disorders of the body, e.g., malabsorption syndrome, coeliac disease, inflammatory bowel disease (e.g., Crohn’s disease), or nephrotic syndrome. It can also result from a specific physiological state with an increased demand (e.g., pregnancy, lactation), or due to excessive loss (e.g., burns, use of copper chelates, penicillamine) [5,6,10,12,21,84]. The most common oxidative damage occurs due to chronic Cu overload or overexposure caused by accidents, occupational hazards, excessive zinc supplementation, or through environmental pollution. Therefore, chronic as well as acute toxicity is very rare [5,7,10,19,20]. However, it should be emphasized that even small changes in Cu concentration are associated with significant toxicity [34], and because of this toxicity, its use as a vomiting agent has been discontinued [5].

Menkes disease (Menkes syndrome) is an inborn, neurodegenerative defect of copper metabolism occurring predominantly in boys, where a lack of copper injections can be fatal [10,19,23]. Girls are mainly carriers of this mutation. Copper is then not absorbed into the child’s body as a result of the genetic abnormality, consequently leading to many serious health problems. One of the characteristic symptoms of this disease is curly, very brittle hair with a steel color, specific facial features, skin lesions, defects of the skeletal, urinary, and vascular systems, and neurological disorders [6,7,22,92]. One of the complications of this disease can be emphysema. This disease is caused by mutations in the P-type ATPase gene [23].

However, the result of over-supplementation and a genetic defect may be Wilson’s disease (an autosomal recessive inherited metabolic disease resulting from alterations in the gene labeled ATP7B on chromosome 13), which mainly involves the inability to remove this pietrichrome from the liver through a defect in the enzyme adenosine triphosphatase. As a consequence, copper accumulates in internal organs, causing deleterious effects also on the kidneys, brain, and nervous system [5,6,7,12,93,94]. Clinical manifestations in chronic Wilson’s disease most commonly include mental changes, motor disturbances (dysarthria, dysphagia, ataxia, writing difficulties), hemolytic anemia, renal tubular dysfunction (uricosuria, hypercalciuria), kidney stones, and fulminant liver failure [12,22]. Its accumulation in the liver is then both pathognomonic and pathogenic, providing a source of cellular reactive oxygen species [13]. The disease usually presents in childhood or adolescence with various neurological syndromes. Meanwhile, Kayser–Fleischer rings are present in the cornea of the eye in the Descemet’s membrane [5,22]. In this case, early treatment with D-pecylamine prevents death and often restores normal liver status [5,7]. In Wilson’s disease, reduced levels (<20 mg/dL) of oxidase-activated ceruloplasmin occur in up to 95% of patients [5].

Environmental exposure to copper and its excessive intake with food are rare in developed countries, but it does occur in developing countries, particularly in India [12]. It is noteworthy that it occurs in rural Indian boys (6 month–5 years), in whom not only a genetic factor is present but there is also an environmental influence (drinking or keeping food in brassware) along with impaired excretion [5,7]. Zn supplementation confirms a beneficial effect, especially in patients with Wilson’s disease [7].

It seems interesting to note that in India, copper is a common suicide agent [5,12]. Meanwhile, spirit water (green water) used very frequently and commonly in African rituals induces hemolysis, kidney failure, or even death after ingestion [5].

## 4. Sources of Copper in Food

The Institute of Medicine, the Food and Nutrition Council, and the Polish Institute of Nutrition recommend a reference intake of Cu for adults of 0.9 mg/day (30 µg/kg body weight per day). The World Health Organization, on the other hand, has set the estimated minimum requirement for Cu at 0.6 mg/day for women and 0.7 mg/day for men. The average daily Cu intake in the USA or Europe (including Poland) is about 1 mg [7,20,21,22,95,96]. The average absorption of Cu from the diet is about 35–50%, and its absorption increases in the case of Cu deficiency in the body [71]. Regarding the NOAEL, EFSA experts assumed a copper dose for adults of 10 mg/day and a UL value of 5 mg/day [96], with a minimum requirement of 0.4–0.8 mg/day [22]. It should be noted that the lethal dose of copper in an untreated adult is about 10–20 g Cu, and in children, it may occur due to accidental ingestion of coins [5,12]. In the USA, the average daily intake of copper is about 1 mg, and its bioavailability from the diet is estimated to be 65–70%, with a biological half-life of about 13–33 days [5]. US guidelines indicate that drinking water should not contain more than 1.3 mg/L [8]. And the Food and Nutrition Board of the Institute of Medicine recommends an age-dependent dietary allowance (RDA) for copper (1–3 years up to 340 µg/day; 4–8 years up to 440 µg/day; 9–13 years up to 890 µg/day; from 14 years up to 900 µg/day) [8].

The richest dietary sources of copper (Table 3) include oysters, beef and calf liver, seafood, chocolate, nuts, legumes, sunflower seeds, pumpkin seeds, red wine, cocoa, potatoes, spinach, vending machine drinks, and whole grains. Copper is also found in green leafy vegetables, avocados, or mushrooms [5,6,7,8,10,12,16,21,22,97].

However, it is worth noting that the range of copper content in foods may vary depending on the food product, its origin, place of manufacture, etc. Examples of the smallest and largest ranges are given below:

- Milk and milk products (0.00 mg/kg—milk drink with whey and cream; 0.40 mg/kg—cheese “Oscypek”);
- Eggs (0.03 mg/kg—chicken eggs white; 0.21 mg/kg—chicken eggs whole dried);
- Meat and meat products (0.00 mg/kg—bone broth; 5.50 mg/kg—calf liver);
- Fish and fish products (0.02 mg/kg—sole raw; 0.33 mg/kg—herring smoked “Pikling”);
- Fats and oils (0.00 mg/kg—oil corn; 0.07 mg/kg—oil olive);
- Cereal products (0.01 mg/kg—corn starch; 0.95 mg/kg—wheat bran);
- Vegetables and vegetable products (0.01 mg/kg—aubergine raw; 1.50 mg/kg—soya beans dried);
- Fruits and fruit products (0.00 mg/kg—strawberries sirup; 0.77 mg/kg—apricots dried);
- Nuts (0.28 mg/kg—walnuts; 1.29 mg/kg—hazelnuts);
- Seeds and grains (0.40 mg/kg—linen seeds; 1.87 mg/kg—sunflower seed dried);
- Sugar and confectionery (0.01 mg/kg—fruit gums; 3.71 mg/kg—cocoa powder);
- Beverages (0.00 mg/kg—black tea infusion average without sugar; 0.09 mg/kg—grape white juice);
- Yeast (0.09 mg/kg—yeast baker’s compressed);
- Other products (0.00 mg/kg—gelatine; 0.29 mg/kg—potato crisps salted);
- Soups (0.01 mg/kg—soup sauerkraut; 0.11 mg/kg—soup green pea);
- Fish dishes (0.06 mg/kg—cod fillets steamed; 0.11 mg/kg—cod fillets breaded and fried);
- Meat dishes (0.01 mg/kg—chicken fillets steamed; 0.57 mg/kg—pork liver sauté fried);
- Vegetable–meat dishes (0.05 mg/kg—sauerkraut with sausage and meat “Bigos” stewed; 0.10 mg/kg—beans baked with meat in tomato sauce);
- Vegetable dishes (0.03 mg/kg—white cabbage salad; 0.54 mg/kg—mushrooms fried);
- Groat, flour, and potato dishes (0.04 mg/kg—pancakes filled with semi-fat fresh cheese fried; 0.25 mg/kg—milled groats boiled with vegetable fat);
- Eggs dishes (0.04 mg/kg—eggs poached with vegetable fat; 0.11 mg/kg—omelet biscuit baked);
- Desserts (0.02 mg/kg—apple compote with sugar; 0.07 mg/kg—strawberries with whipped cream);
- Other dishes (0.02 mg/kg—cheese Tilsit paste; 0.10 mg/kg—fresh cheese and fish smoked paste) [97].

Natural water contains 4–10 mg Cu/L, while potable water represents 6–13% of the average daily requirement. The human body is able to control excessive amounts of Cu in the body by decreased absorption or increased excretion [12,98]. As water naturally contains copper, the excess is most often the result of its release from old pipes, household taps, standing water, or hot water (it dissolves easily at high temperatures) [6].

Absorption of copper facilitates its binding to several essential amino acids (Cys, His, Asp, Met, Tyr, Gly, and Thr). Its presence in the diet also facilitates binding as ligands to organic acids (gluconic, lactic, citric, and acetic acids) [6]. It is worth noting that Cu is able to catalyze the formation of ROS, and nutrients with antioxidant properties may provide protection against the induction of this element into oxidative damage [7,12,17,18].

Vitamin E may prevent endothelial dysfunction associated with cholesterol metabolism, and vitamin E supplementation alone may be beneficial in preventing ROS-induced functional impairment in atherosclerosis [7]. Meanwhile, vitamin C (an antioxidant) can reduce metals with redox activity, such as Cu and Fe, increasing the pro-oxidant activity of these metals. Also, it can serve as a pro-oxidant (at low concentrations) as well as an antioxidant (at high concentrations) in vitro. Ascorbic acid enhances the cytotoxic effect of Cu ions in human embryonic cells (CLV102 and Lu106) and in exposed human melanoma cells (Mel8). However, it should be emphasized that ascorbic acid from the diet, as well as from supplementation, protects against Cu toxicity and absorption in tissues, mainly through reduced absorption in the small intestine [6,7,12]. Also, a well-balanced diet rich in Se can protect against a toxic version of Cu. This occurs due to the antioxidant properties of Se, which owe their activity to the selenoenzymes in proteins. Meanwhile, Se supplementation does not protect Cu-induced liver damage [7,12]. It is also worth noting that another beneficial effect on oxidative stress levels is the action of Zn, as part of the Cu enzyme Zn-SOD, in which Cu and Zn have a strongly antagonistic effect. High concentrations of Zn prevent absorption in the intestine, liver, and kidneys through the formation of metallothionein. Zinc also helps to reduce the formation of free radicals where different types of Cu bonds are formed, competing with Cu for a place in the molecule. Zn then binds to the substrate in question, probably histidine, stopping the formation of free radicals, and Cu is displaced [7,20]. Interestingly, red wine components (polyphenols) have been shown to prevent LDL oxidation and Cu-dependent and Cu-independent oxidation of LDL and HDL proteins and lipids in a dose-dependent manner [6,7,22]. As shown by Se, beta-carotene, alpha-lipoic acid, and polyphenols may provide protection against Cu-induced oxidative damage, respectively, although this requires further in vivo studies [7].

## 5. Copper in the Environment

The natural concentration of this element in soil is about 50 ppm (2–250 ppm) Cu, but it is also released into the air from natural sources (e.g., windblown dust, volcanoes, forest fires, decaying plants, sea spray) or from artificial sources (e.g., iron production, steel production, municipal incinerators), or as a result of human activities (e.g., landfills, wood production, phosphate fertilizers). In the atmosphere alone, it ranges from 5 to 200 ng Cu/m^3^, while in the vicinity of smelters, it can reach up to 5000 ng/m^3^ [5,7,8]. In turn, its emissions to the environment represent 0.3%, coming mostly from natural sources (wind-borne, dust, volcanoes, forest fires, sea spray), as well as anthropogenic sources (nonferrous metal production, copper smelters, iron and steel production, municipal incinerators) [5,8]. Copper deposits also occur in sedimentary and volcanic rocks in a variety of geological environments, and the resulting deposits can be either layered, massive, or dispersed [3,7,20,99]. It is estimated that municipal wastewater treatment plants remove up to 80% of total copper from the water during pretreatment, where concentrations can be <100 μg Cu/L in the effluent. Water that remains overnight in plumbing fixtures can contain up to 60 mg Cu/L (20–75 ppb). With a taste threshold, a value of 1–5 mg Cu/L is perceptible, and a slight blue-green coloration appears in the water at levels > 5 mg Cu/L [5,8]. Meanwhile, copper concentrations in rivers or lakes range from 0.5 to 1000 ppb, with some groundwater up to 2800 ppb [8].

Almost as much copper is recovered from recycled materials each year as is mined, which contributes to its continually high value in the premium scrap classification. Thanks to its renewability, it is recognized as a sustainable material, i.e., consequently environmentally friendly [4,5].

Copper has always been, is, and will continue to be one of the most important metals traded internationally, and its global trend is confirmed by its largest share in metallurgical products (over 15%) [3,100]. It is well dispersed globally and available, with its supply distributed among the world’s top four producers: Chile (28%), Peru (12%), China (9%), and the United States (7%) [4,101]. Meanwhile, in 2020, Poland (35 million tons) was ranked 7th among countries with the largest copper reserves. Chile (200 million tons), Australia and Peru (87 million tons each), Russia (61 million tons), Mexico (53 million tons), and the USA (51 million tons) produce the most, followed by Poland with Indonesia (28 million tons), China (26 million tons), Congo (19 million tons), and the rest together (220 million tons) [102]. Utah (USA) is home to the deepest open-pit copper mine in the world (1200 meters deep, 4 km in diameter) made by human beings [3,100]. All the information collected for some time on copper resources confirms its abundance. It should also be noted that most of this element is mined by large, international companies adhering to strict safety and environmental standards. A mixture of well-known open-pit and underground mining technologies is then used. Primary copper demand in the EU is about 4.2 million tons per year, of which 37% is met through imports, 18% through European copper production (mainly Poland and Sweden), and about 45% through recycling. Thus, the EU has a fairly manageable exposure to its imports and is at the forefront of the circular economy [4,101]. Poland extracted 33.8 billion tons of Cu in 2022 from deep mines (Lubin, Rudna—the largest in Europe, Polkowice). Its largest accumulation in Poland is associated with deposits located on the pre-Sudetic monocline (the New Copper Belt) and in the North Sudetic Basin (the Old Copper Belt), formed about 200 million years ago [3,100]. The most common destinations for Polish electrolytic copper in 2018 included Spain (150 t), Germany (92 t), China (90 t), Italy (33 t), France (31 t), Slovakia (15.6 t), Turkey (14.8 t), Japan (5.3 t), and India (4.5 t) [102].

It is also worth noting that as a biocide (especially for Gram+ and Gram− bacteria, fungi, viruses, and other bacterial spores), copper biocides have become indispensable worldwide. Many thousands of tons per year are used in agriculture, wood preservation, antifouling paints, and the elimination of unpleasant odors from textiles [9].

## 6. Source of Copper in Different Materials

Cu comes from Cyprus, where this field precious metal was mined around 2000 BC. Although the first traces of its production and processing were already recorded more than 5,000 years BC in Mesopotamia, the Sinai Peninsula, and the Mediterranean countries. It is worth noting that copper itself forms more than 150 different minerals. Its largest sources are minerals such as chalcopyrite and bornite, and it is obtained by smelting, leaching, and electrolysis. At room temperature, it is resistant to dry air, while it reacts with oxygen. When heated, it can dissolve in oxidizing acids. Meanwhile, in humid conditions, it becomes covered with a patina, or is also used for decorative purposes. Copper also forms complex compounds, and its aqueous solutions of compounds are blue in color. Its soluble compounds are mainly poisonous. Their main use is as an insecticide or fungicide (CuSO_4_, Cu, O), or for the impregnation of wood (pigments, paint catalysts: CuO, CuCO_3_, Cu(OH)_2_.) [2,6]. Noteworthy is the fact that it is the main alloying component of brasses (Cu-Zn), new silver with the addition also of the alloys of some steels, bronzes (Cu-Sn), cast iron, cast steel, aluminum and bearing alloys, zinc, gold, and silver, and binders for joining metals, as well as dental alloys. Copper also guarantees temperature and pressure stability, as well as an aesthetic appearance [1,2,3,4]. 

Because of its distinctive properties, copper is considered a very good material for its widespread use, including its resistance to corrosion (which guarantees its longevity), its biostatic effect (inhibiting the growth of bacteria in the production of doorknobs, kitchen worktops, and tables), and it is nonmagnetic [2,4]. 

Copper itself is widely used in, among other things,

- roofing, gutters, building fittings, door fittings, locks, architectural decorative elements (also interior design);
- all kinds of installations (electrical, heating, gas, fire-fighting, air-conditioning sprinklers, air conditioners, lighting, heat exchangers, parts of rocket engines, wireless chargers);
- in the hydraulic area, plumbing (cold and hot water pipes, taps);
- in the chemical and food industry apparatus (e.g., coolers, rectification columns, chemical and distillation apparatus, etc.);
- electrical machine components and printed circuits, microelectronic material technology itself as well as optoelectronic materials (solar cells, electro-acoustic relays, batteries);
- in the electrical and energy sector (electrical generators, transformers, wind power stations, types of cabling, wires, sockets);
- in the telecommunications industry (computer connectors, computer chips, graphics cards, hard drives, cables, mobile phones, computers, televisions, household appliances);
- in the automotive industry (trains, aircraft, trucks and cars, including electric vehicles) or mechanical engineering (e.g., sealing of oil injectors);
- in wind and solar energy (supports the proper functioning and efficiency of wind turbines, protects the generator, grounds towers against lightning strikes, conducts electricity);
- in the military sector (weapons, transport);
- in the medical sector (dental products—dental bridges/crowns, intrauterine contraceptive devices—100–150 mg; folk spirit water—13g/L);
- in the mint (Polish one-penny coins are made from manganese brass, containing 59% copper, 40% zinc, 1% manganese);
- in the chemical industry (production of pesticides);
- copper compounds are used in agriculture to treat diseased plants (e.g., molds), to treat water, and as a preservative for wood (impregnation), leather, and textiles [1,2,3,4,5,6,7,8,9].

## 7. Prevention

In addition to the abovementioned noted symptoms, through a typical physical examination and history, it is necessary to determine blood levels of total copper and the free fraction, as well as levels of ceruloplasmin (copper transport protein). The determination of copper levels in daily urine collections and blood levels of superoxide dismutase and cytochrome c oxidase are then recommended [5,20,22,92]. A tight regulation of copper metabolism is required to ensure sufficient copper availability for the necessary enzymes, without toxic effects caused by an excess of this element. Knowledge of its regulation allows us to consider a new area of potential therapeutic interventions based on copper supplementation or removal in neurodegenerative diseases, including Wilson’s disease, Menkes disease, Alzheimer’s disease, Parkinson’s disease, and others [5,6,13,20,50,91]. Additional information on copper ions and also on tumor immunity itself may broaden the knowledge on new therapeutic approaches for cancer patients and improve their clinical prognosis [19].

It is recommended to drink only cold water from taps and to use only cold, longer-flowing water from the source for cooking [6,71,72]. It is therefore advisable to drain the water for about 15–30 s to reduce its content [8].

It is worth noting that there are no well-designed studies of workers exposed to copper (e.g., smelters, metallurgy, mechanical industry, etc.) that demonstrate chronic toxicity, acting particularly on the respiratory tract and gastrointestinal tract. There are also currently no data confirming a safe and biological limit in workers exposed to copper. It is only recommended that workers be controlled for copper vapor of 0.2 mg Cu/m^3^ and copper dusts and mists of 1 mg Cu/m^3^ [5,8].

According to Focarelli et al. [11], bacterial copper detoxification systems may be viable targets for future development of new antibiotic development programs [9]. There is also one novel strategy for treating people with emphysema, through the use of topical supplementation, which would then restore copper levels in the lungs, also improving elastin. Although, in this case, it is important to use high doses, which can be problematic due to entering the circulatory system quickly, thus potentially affecting the brain or liver toxically [23].

Recently discovered and introduced copper nanoparticles even have ideal antibacterial activity against *Staphylococcus aureus* (including methicillin-resistant *S. aureus*), *Bacillus subtilis*, *Proteus vulgaris*, and *Escherichia coli* [50,79]. The characteristics of copper nanomaterials (i.e., antibacterial/antifungal) and the ability to accelerate the healing of infected wounds have enabled applications to other medical fields, such as dentistry. They can be added to dental adhesives, tooth-filling materials, implant coatings, orthodontic abutment coatings, or denture materials [39,50,103].

Thanks to the use of copper oxygen molecules in polymer carriers and their strong biocidal effect, they are now being used in health-promoting as well as consumer products, for example, in the production of hospital linen, nurses’ garments, patients’ dressing gowns and pajamas, protective breathing masks, wrinkle-reducing pillowcases, socks for the prevention and treatment of foot fungus (athlete’s foot) and to reduce the risk of skin pathologies in diabetics, and wound dressings to reduce dressing and wound contamination and promote wound healing [9].

Copper content in biological or environmental samples is mainly determined using atomic absorption spectrometry (flame or graphite) and inductively coupled plasma atomic emission spectroscopy. In the absence of this equipment in the laboratory, rapid colorimetric tests with detection ranges close to 0.1 mg Cu/L are available [80]. As the tests are not available in physicians’ offices, samples must be sent to a special laboratory [8].

Only in the case of acute poisoning is the use of chelating agents recommended, abandoning the use of vomiting agents or laxatives. And after, the ingestion of drugs with high levels of CaNa2EDTA or intramuscular BAL is recommended. For Wilson’s disease, the treatment of symptomatic neurological, psychiatric, and liver disease with d-penicillamine chelation is recommended. Foods with a high concentration of copper should also be avoided (shellfish, nuts, liver, mushrooms, chocolate) [5,12].

Examples of preventive interventions:

- governments and major institutions in the country should regularly check Cu levels in food, soil, and air;
- continuously monitor drinkable water systems;
- conduct regular corrosion control, servicing, and regular testing;
- secure drinking sources (especially in schools) with appropriate filtration systems;
- remove old drains, pipes, and sanitation (e.g., taps) from service and replace with new ones;
- use cold water, drained for a minimum of 1 min;
- use appropriate posting if water cannot be consumed in the chosen location (e.g., “Do not use”);
- wash hands regularly;
- replace kitchen appliances with glass/porcelain ones;
- use of water treatment technology [39,97,104,105,106].

## 8. Conclusions

Undoubtedly, copper is essential for brain cells (as a cofactor and component of many enzymes involved in biochemical pathways); however, excess amounts are harmful. A high concentration of Cu can contribute to many diseases, including neurodegenerative, cardiovascular, immune, bone, respiratory, reproductive, gastrointestinal, skin, and even some cancers. Cu is essential, and in optimal doses provides antioxidant protection, and its deficiency reduces the body’s ability to cope with oxidative stress, which is a consequence of many serious diseases. Being involved in a tremendously wide range of many biochemical reactions, providing or accepting electrons, Cu ions also bind to various proteins or enzymes as cofactors or structural components. It is involved in the most important physiological proxies, such as energy metabolism, mitochondrial respiration, inflammatory reactions, and antioxidants, so it is worth focusing attention on it. The development of civilization and the continued widespread use of Cu in all our electrical devices will not be stopped, but the health of those directly involved in its extraction, production, or distribution can be controlled. It is extremely difficult to assess the effects of copper on the human body because of its ubiquity and the increasing reports in the literature about its effects, including copper nanoparticles.

Several factors influence the retention or bioavailability of copper from diet alone, i.e., age, amount and form of copper in the diet, and genetic background. Further research is still needed to better understand how much and to what extent copper affects the health of the body of the current generation, which seems to be already familiar with this element.

## Figures and Tables

**Table 1:** Summary of the effect of copper on individual parts of the human body [6,9,11,13,14,16,17,18,19,22,24,25,26,27,28,29,30,31,32,33].

Human Body	Effects of Exposure to Copper
Immunological system	- occurs in numerous metalloenzymes - has a protective effect on DNA, proteins, or lipids - can catalyze the production of hydroxyl radicals - influences the development and function of the immune system (humoral and cellular and production of immunoreactive substances) - when deficient, can increase host susceptibility to various pathogens - its antibacterial toxicity is used by phagocytic cells to kill pathogens that accumulate at sites of infection
Skeletal system	- is involved in strengthening connective tissue structures, mainly bone - stimulates the differentiation of mesenchymal stem cells toward the osteogenic lineage
Circulatory system	- supports cardiac and vascular function (e.g., through the synthesis of red blood cells) - improves blood morphotic parameters - minimizes the risk of atherosclerotic lesions - severe intoxication with copper sulphate can cause, among other things, intravascular hemolysis - when it accumulates in the heart muscle, it can adversely affect its function
Nervous system	- supports normal brain development - helps eliminate harmful oxygen “free radicals” - has a protective effect on the reduction in beta amyloid levels in the cerebrospinal fluid - controls gene expression in the nucleus accumbens - is involved in myelination of the brain - stimulates brain function and modulates neural transmission - has a positive effect on cognitive functions - participates in the synthesis of ATP - low intake may be associated with mental retardation or impaired central nervous system function
Digestive system	- chronic toxicity affects the liver (cirrhosis with episodes of hemolysis), renal tubular damage, gastritis - partially absorbed in the stomach, duodenum, and ileum - ingestion of concentrations >30 ppm Cu causes gastrointestinal irritation (nausea, vomiting, abdominal pain), and ≥25 mg Cu/L is associated with acute gastroenteritis - renal impairment is mild (e.g., anuria or scanty uremia)
Respiratory system	- occurrence of fever caused by metal fumes (chills, muscle aches, headache, malaise, dry throat) - large vapors irritate the airways (mucous membranes of the mouth, eyes, or nose), respiratory tract inflammation, or cause perforation of the nasal septum
Reproductive system	- can potentially affect reduced fertility - in correlation with other elements (e.g., zinc, selenium) is important for maintaining reproductive health in both sexes - essential for the production of male gametes (role in cell division processes–mitotic and meiotic) - deficiencies as well as excesses cause reduced fertility in men
Skin	- component of tyrosinase, a metalloenzyme involved in the synthesis of melanin (skin and hair pigment) - prevents premature greying of hair, alleviates symptoms of vitiligo–protective effect against harmful UV radiation - participation in the synthesis of collagen and elastin - reduction in fine lines and wrinkles on the face - accelerates wound healing - improves the general condition of the skin - deficiencies result in loss of skin, epidermis, or change in hair color - used in face creams (e.g., anti-aging creams) - in excess, causes inflammation, redness, contact eczema, itching
Cancers	- used in cancer therapy by inhibiting, among other things, the activity of the p53 protein - inhibits the growth and proliferation of cancer cells themselves, their metastasis and angiogenesis - there is a correlation between high Cu levels and the metabolic demand of prostate, breast, colon, liver, colorectal, and brain tumors - copper ions are involved in the development, growth, and metastasis of tumors through various pathways - regulates PD-L1 expression by inducing an antitumor immune response

**Table 2:** The most common ailments resulting from copper deficiency and excess in the body [5,6,7,10,13,22,84,92,93].

Copper Deficiency	Copper Excess
anemia Menkes disease numbness in the limbs bone weakness muscle weakness trouble concentrating decreased appetite increased infections lipid profile disorders heart arrythmia increase in blood pressure optic neuritis deterioration of the skin condition hair loss and premature graying hypothyroidism	muscle stiffness involuntary facial grimaces and body movements lower abdominal pain, ascites, and jaundice developing due to liver cirrhosis aggressive disorders, psychosis cardiovascular breakdowns drooling lethargy, coma copper deposits in the cornea of the eye, visible as a brown ring death

**Table 3:** Threshold copper content in selected food products in Poland [97].

Food Product	Threshold Content (mg/kg Fresh Matter)
Milk and milk products	0.00–0.40
Eggs	0.03–0.21
Meat and meat products	0.00–5.50
Fish and fish products	0.02–0.33
Fats and oils	0.00–0.07
Cereal products	0.01–0.95
Vegetables and vegetable products	0.01–1.50
Fruits and fruit products	0.00–0.77
Nuts	0.28–1.29
Seeds and grains	0.40–1.87
Sugar and confectionery	0.01–3.71
Beverages	0.00–0.09
Yeast	0.09
Other products	0.00–0.29
Soups	0.01–0.11
Fish dishes	0.06-.011
Meat dishes	0.01–0.57
Vegetable–meat dishes	0.05–0.10
Vegetable dishes	0.03–0.54
Groat, flour, and potato dishes	0.04–0.25
Eggs dishes	0.04–0.11
Desserts	0.02–0.07
Other dishes	0.02–0.10

## Data Availability

Not applicable.
